# Serum protein electrophoresis in healthy and injured southern white rhinoceros (*Ceratotherium simum simum*)

**DOI:** 10.1371/journal.pone.0200347

**Published:** 2018-07-25

**Authors:** Emma H. Hooijberg, Michele Miller, Carolyn Cray, Peter Buss, Gerhard Steenkamp, Amelia Goddard

**Affiliations:** 1 Department of Companion Animal Clinical Studies, Faculty of Veterinary Science, University of Pretoria, Pretoria, South Africa; 2 Department of Science and Technology/National Research Foundation Centre of Excellence for Biomedical TB Research/Medical Research Council Centre for Tuberculosis Research, Division of Molecular Biology and Human Genetics, Faculty of Medicine and Health Sciences, Stellenbosch University, Stellenbosch, South Africa; 3 Department of Pathology & Laboratory Medicine, University of Miami Miller School of Medicine, Miami, Florida, United States of America; 4 Veterinary Wildlife Services, South African National Parks, Kruger National Park, Skukuza, South Africa; Università degli Studi di Milano, ITALY

## Abstract

Investigation of globulin fractions by serum protein electrophoresis (SPE) is the first step towards evaluation of the proteome in the southern white rhinoceros (*Ceratotherium simum simum*). Furthermore, identification of changes in globulins in animals with poaching and other injuries can guide discovery of potentially useful biomarkers of inflammation. The aim of this study was to develop reference intervals for agarose gel SPE in healthy white rhinoceros and to compare these serum protein electrophoresis results to those from animals with tissue trauma. Reference intervals for total serum protein and agarose gel electrophoretic albumin and globulin fractions were generated using serum samples from 49 healthy free-ranging adult white rhinoceros. A standardised gating system together with identification of specific proteins by mass spectrometry aided in fraction identification. Six globulin fractions were identified: α1a, α1b, α2, β1, β2 and γ. Reference intervals were generated for total serum protein (76–111 g/L), albumin (10–27 g/L) and globulin fractions (α1a: 1.6–3.2 g/L; α1b: 1.7–3.6 g/L; α2: 16.1–26.6 g/L; β1: 6.6–18.2 g/L; β2: 11.8–30.4 g/L; γ: 10.4–23.1 g/L; albumin: globulin ratio: 0.12–0.39). Results were compared to those from 30 animals with various degrees and chronicities of tissue trauma. Wounded animals had lower concentrations of total serum protein, albumin, total globulin, α and β1 globulins, lower percentages of α2 and β1 globulins, and higher percentages of β2 and γ globulins. These protein changes are similar to those seen in human patients with wounds rather than classic acute phase or chronic inflammatory responses.

## Introduction

The southern white rhinoceros, *Ceratotherium simum simum* (referred to hereafter as the white rhinoceros), is heavily poached in southern Africa, with over 4 000 animals killed in South Africa between 2013 and the end of 2016 [1]. These figures do not include those animals which survive a poaching attempt and are subsequently treated by veterinarians. For example, 54 injured rhinoceros were treated by South African National Parks veterinarians from 2014–2016, 31 of which were ultimately euthanized due to their injuries [2]. At least 38 orphan rhinoceros calves were being cared for in South Africa at the end of 2016, many of which required veterinary care during their rehabilitation process [1].

Clinical pathology plays an important role in the diagnosis and monitoring of animal disease, and clinical chemistry reference intervals for the white rhinoceros for various analytical methods have been recently published [3, 4]. Noteworthy in these recent, as well as older studies, is the high concentration of serum or plasma total protein (particularly globulins) and low concentration of albumin in this species [4]. Mean concentrations reported for total serum or plasma protein range from 76–101 g/L, albumin from 25–28 g/L and globulins from 66–77 g/L [3–8]. In comparison, means or medians reported for the black rhinoceros, *Diceros bicornis*, are 81–95 g/L for total serum protein (TSP), 35–36 g/L for albumin and 46–54 g/L for globulins [5, 9, 10]. Median values for TSP of 81 g/L, albumin of 39 g/L and globulin of 39 g/L have been published for Sumatran rhinoceros (*Dicerorhinus sumatrensis*) [11].

Serum protein electrophoresis (SPE) is considered the reference standard for the determination of albumin and globulin [12]. Globulins consist of hundreds of different proteins, which migrate into defined fractions, conventionally grouped as α-, β-, and γ-globulins. The α-globulin group contains positive acute phase proteins, the β-globulins include transferrin, complement factors, lipoproteins and some immunoglobulins. The majority of immunoglobulins have been described to migrate to the γ- fraction in many species, including other Perissodactyls like the horse [13–16]. Variations in serum proteins with concentrations above 0.5 g/L may result in changes to the electrophoretic pattern and concentrations of the various fractions. A decrease in albumin and increases in the α2-globulins, α2-macroglobulin and haptoglobin, occur typically in acute inflammation as part of the acute phase response, while a polyclonal increase in immunoglobulins (β2 and γ fractions) occurs with chronic inflammation [16, 17]. White rhinoceros with tissue trauma caused by gunshot wounds or fighting may sustain damage to both soft tissue structures and bone. These animals necessarily have either an acute or chronic, localised or systemic inflammation. Wounds are associated with a catabolic state, and protein demand can be increased by 250% in human wound patients as protein is needed in every phase of wound healing [18]. Differences in serum protein concentrations and composition between injured and healthy white rhinoceros may be diagnostically and prognostically useful.

Four previous studies, performed between 1976 and 1994, reported results of cellulose acetate membrane SPE in healthy white rhinoceros [6–8, 19]. Only one of these studies describes and displays an electrophoretogram [7]. Cellulose acetate membrane has been replaced by agarose gel for SPE; migration patterns are not identical with these two matrices, and information for agarose gel SPE for the white rhinoceros is lacking [20]. In addition, SPE changes have not been investigated in injured white rhinoceros.

The first objective of this study was to generate reference intervals for TSP and agarose gel SPE fractions in healthy white rhinoceros. The second objective was to compare protein values from white rhinoceros with tissue trauma to the healthy group in order to identify clinically relevant changes.

## Materials and methods

### Study population and samples

The reference sample group consisted of 50 adult free-ranging white rhinoceros of both sexes from the Kruger National Park in South Africa (23°49ʹ60ʺS, 31°30ʹ0ʺE). These animals were immobilized primarily for management or translocation purposes. Immobilization was performed according to the South African National Parks Animal Use and Care Committee approved Standard Operating Procedure for the Capture, Transport and Maintenance in Holding Facilities of Wildlife. The immobilization protocol used for these animals has been fully described elsewhere [4]. Blood sampling took place within 15 minutes of immobilization. Blood was collected directly into a serum vacuum collection tube (Greiner Bio-One, Lasec S.A., PTY LTD Cape Town, 7405, South Africa) from an auricular vein. Samples were placed upright to clot in a cooler bag with ice blocks and processed within three hours of collection. Sample tubes were centrifuged at 1300 *g* for 10 minutes, and serum aliquoted into cryotubes (Greiner Bio-One, Lasec S.A., PTY LTD Cape Town, 7405, South Africa) and frozen for 22 months at -80°C.

A physical examination was carried out on immobilized animals, and those showing no abnormalities were considered to be healthy. Age was estimated from horn and body size and animals were categorized as calves (< 2.5 years), subadults (2.5–7.0 years) and adults (> 7.0 years) [3, 21]. White rhinoceros estimated to be less than seven years of age or exhibiting bullet or dehorning wounds or any other abnormalities were excluded from the reference sample group.

Serum samples from 30 white rhinoceros with tissue trauma were also analyzed. Injured animals were further subdivided in those having acute (duration of injury ≤ two days) or chronic injuries, when this information was available in clinical records. Twenty-three of these animals, including two calves, were from the Kruger National Park, and samples were collected using the protocol described above. These samples were frozen at -80°C for 6–27 months. Four samples were received by the clinical pathology laboratory of the Onderstepoort Veterinary Academic Hospital (OVAH) as part of another research project investigating injured white rhinoceros. The immobilization protocol and sampling conditions for these four individuals were unknown. Samples from three white rhinoceros calves that were treated as inpatients in the OVAH were also included. These samples were collected without immobilization from the auricular vein. For these last seven individuals, serum was received by the laboratory in serum vacuum tubes which were left to clot for 30 min and centrifuged at 2100 *g* for 8 minutes. The serum was aliquoted and frozen at -20°C for 6–8 months for the calves, and at -80°C for 28–36 months for the other four adults.

Samples originating from the Kruger National Park were subsequently transported frozen on ice to the clinical pathology laboratory at the OVAH and again stored at -80°C. Most samples from injured animals were also used for other projects and so were subjected to at least one additional thaw-freeze-thaw cycle. Samples were excluded if gross hemolysis, lipemia or icterus was present.

Before analysis, batches of samples were left to thaw at room temperature, mixed and centrifuged at 2100 *g* for 8 minutes.

### Sample analysis

#### Total serum protein

TSP was measured by the biuret reaction on an automated wet chemistry analyzer, the Cobas Integra 400 Plus (Roche Products (Pty) Ltd, Basel, Switzerland). Maintenance of the analyzer was carried out according to the manufacturer’s guidelines and assay performance was monitored by daily internal quality control and monthly external quality control according to laboratory protocols.

#### Electrophoresis

Electrophoresis was performed on split beta agarose gel using the automated Interlab Pretty platform (Interlab S.r.L., Rome, Italy) according to the manufacturer’s instructions. Required sample volume was 30 μL with places for 13 samples on each gel. A voltage of 400 V was applied for eight minutes, followed by fixation and staining with acid blue stain. After gels were dried, they were placed on a flat-bed scanner for densitometric analysis. Results were displayed using the software program Elfolab (Interlab S.r.L., Rome, Italy). Protein fractions were identified on both the resulting electrophoretogram and visually on the gel. Fractions were separated using a standardized method using relative migration distances (Rf), whereby the relative distance of the mid-point of each peak (in mm) compared to the mid-point of the albumin peak (in mm), was kept as constant as possible [22]. Gating and naming of fractions proceeded as follows: the first anodal peak was identified as albumin and the first gate placed in the trough cathodal to this peak; the gate discriminating between α- and β-globulins was placed in the midpoint of the tracing, in the small trough between the two peaks directly anodal and cathodal to the midpoint; α-globulins were further subdivided into two small α1 fractions and one large α2 fraction based on the presence of three peaks; β1 globulins were identified as the first peak after the α2-β gate; β2 –globulins as the next peak; the β-γ gate was placed on a notch cathodal to the β2 peak. For verification, the Rfs of each of these fractions was compared and found to be similar to those from cats, dogs, horses, cattle and sheep [22]. The relative protein concentration in each fraction was multiplied by the TSP concentration determined by the biuret method to give the absolute protein concentration in each fraction.

Intra-gel imprecision was evaluated by running one sample in all 13 places on one gel, and inter-gel imprecision was evaluated by running this same sample in position 1 on eight different gels over a number of days. Three aliquots of the same sample were used for the latter experiment. Each of these aliquots was stored for up to 48 hours at 4°C after thawing. All samples were analysed by one operator (EHH) within 24 hours of automated total protein and albumin measurement.

#### Confirmation of α-globulin designation

In order to confirm that the third globulin peak was α2 and not β1, the bands designated as α1b and α2 were further analysed with the specific aim of identifying proteins known to migrate into these fractions. Specimens of each band from four different lanes were excised from the gel used for the intra-gel imprecision study. Sample preparation and proteomic analysis, as described below, was performed by the Proteomics Unit, Central Analytical Facility, Stellenbosch University, Stellenbosch, South Africa. The methods used for protein extraction, liquid chromatography (LC) and mass spectrometry (MS) are summarised below. Full details can be found in S1 Text.

Bands were destained with 10% acetonitrile in 100 mM Tris pH 8 before reduction with 2 mM triscarboxyethyl phosphine in 100 mM NH_4_HCO_3_ and then washed with 100 mM NH_4_HCO_3_. Proteins were digested by rehydrating the gel pieces in trypsin solution and incubating at 37 °C overnight. Peptides were extracted from the gel pieces once with 50 μL water and once with 50% acetonitrile. Residual digest reagents were removed using an in-house manufactured C18 stage tip. The resulting bound sample was washed with 30 μL of an aqueous solution containing 2% acetonitrile and 0.1% TFA before elution with 30 μL of an aqueous solution containing 50% acetonitrile and 0.05% TFA. The eluate was evaporated to dryness. The dried peptides were dissolved in an aqueous solution containing 2% acetonitrile and 0.1% FA for LC-MS analysis. LC was performed on a Thermo Scientific Ultimate 3000 RSLC (Thermo Fisher Scientific) equipped with a 2 cm x 100 μm C18 trap column and a 35 cm x 75 μm in-house manufactured C18 analytical column (Aeris C18, 3.6 μm; Phenomenex, Torrance, CA, USA). For the solvent system, Solvent A (the loading solvent) consisted of 2% aqueous acetonitrile with 0.1% FA and Solvent B consisted of 100% aqueous acetonitrile. MS was performed using a Thermo Scientific Fusion mass spectrometer (Thermo Fischer Scientific) equipped with a Nanospray Flex ionization source. The raw files generated by the mass spectrometer were imported into SearchGUI (Compomics, Ghent, Belgium) and the X!Tandem algorithm was selected. Database interrogation was performed against a concatenated database created using the Uniprot [23] order Perissodactyla database and a contaminant database with semi-tryptic cleavage allowing for 2 missed cleavages. Precursor mass tolerance was set to 10 ppm and fragment mass tolerance set to 0.02 Da. Protein deamidation (NQ) and oxidation (M) were allowed as dynamic modifications. The msf file output from Proteome Discoverer was imported into Scaffold Q+ (version 4.4.6, Proteome Software Inc., Portland, OR) and additional validation performed. Proteins were considered to be positively identified if the protein identification probability was 100%.

### Data analysis

#### Reference intervals

Calculation of 95% reference intervals for TSP, SPE albumin and SPE globulin fractions was performed using Reference Value Advisor (RefVal) version 2.1, according to published guidelines for veterinary species [24, 25]. Histograms of the data were inspected visually. Dixon and Tukey tests were used to identify outliers, and the Anderson-Darling and McWilliams runs tests used to assess normality and symmetry, respectively. A *P-*value of < 0.27 was used with the Anderson-Darling test to increase specificity [26]; *P* was set at < 0.05 for the runs test. Box-Cox transformation was applied to non-Gaussian data. The robust method was used for reference limit determination on native or transformed normally distributed data sets. If data were not normally distributed, the non-parametric method was applied. The 90% confidence interval (CI) of the limits was calculated using a non-parametric bootstrap method [27]. Results were compared for all fractions for females versus males using the Mann-Whitney U test.

#### Comparison of healthy to injured animals

All of the following statistics were applied to the injured group as a whole, and to the acutely and chronically injured subgroups. Descriptive statistics were performed on data for TSP, SPE albumin and SPE globulin fractions. The Shapiro-Wilk test with *P* < 0.05 was used to assess normality. Results from injured white rhinoceros were compared to those from healthy animals, using either the Mann-Whitney (non-parametric data) or *t*-test (parametric data) (*P* < 0.05). The frequency of results from injured animals that were outside of the reference interval was calculated for each analyte. Statistical analysis was performed using MedCalc for Windows, version 17.6 (MedCalc Software, Ostend, Belgium).

Ethical approval specifically for this study protocol was granted by the University of Pretoria Animal Ethics Committee (applicable certificate numbers V042-15, V011-17). The Faculty of Veterinary Science, University of Pretoria is authorized to work with white rhinoceros under the terms of Threatened or Protected Species (TOPS) Standing Permits S02655, S03007, S03013. South African National Parks is authorized to work with white rhinoceros under the terms of TOPS Standing Permits S21201, S03317, and S07620. TOPS permits are issued by the Department of Environmental Affairs, Government of the Republic of South Africa.

## Results

### Study population

The reference sample group consisted of 50 adult white rhinoceros, inclusive of 25 males and 25 females. These animals were found in the southern part of the Kruger National Park, from the southern park boundary up to the Tshokwane area. One male, most likely dehydrated, was excluded from the reference interval calculations because of a high albumin concentration, which was identified as an outlier by both Tukey and Dixon tests. Signalment and full clinical information for the 30 injured white rhinoceros is presented in S1 Table, with a summary of this information presented in Table 1. The chronicity of injury was estimated for 28 animals. Two samples were available for two of the calves from the OVAH.

**Table 1:** Summarized signalment and clinical information for 30 white rhinoceros with tissue trauma.

**Life stage**	23 adults 2 subadults 5 calves
**Sex**	18 males 12 females
**Origin**	23 KNP 4 external 3 OVAH
**Clinical findings**	14 with bullet wound only 2 with bullet and other wounds 3 with fight wounds 2 with dehorning wound 2 with surgical wound 7 with wounds of unspecified origin
**Chronicity**	11 acute 17 chronic 2 unknown

KNP: Kruger National Park; SA: South Africa; OVAH: Onderstepoort Veterinary Academic Hospital

### Sample analysis

#### Total protein

The TSP assay met analytical performance goals over the course of the study. Estimates of imprecision (CV) derived from internal quality control results were 2.1%.

#### Electrophoresis

**Fig 1:**
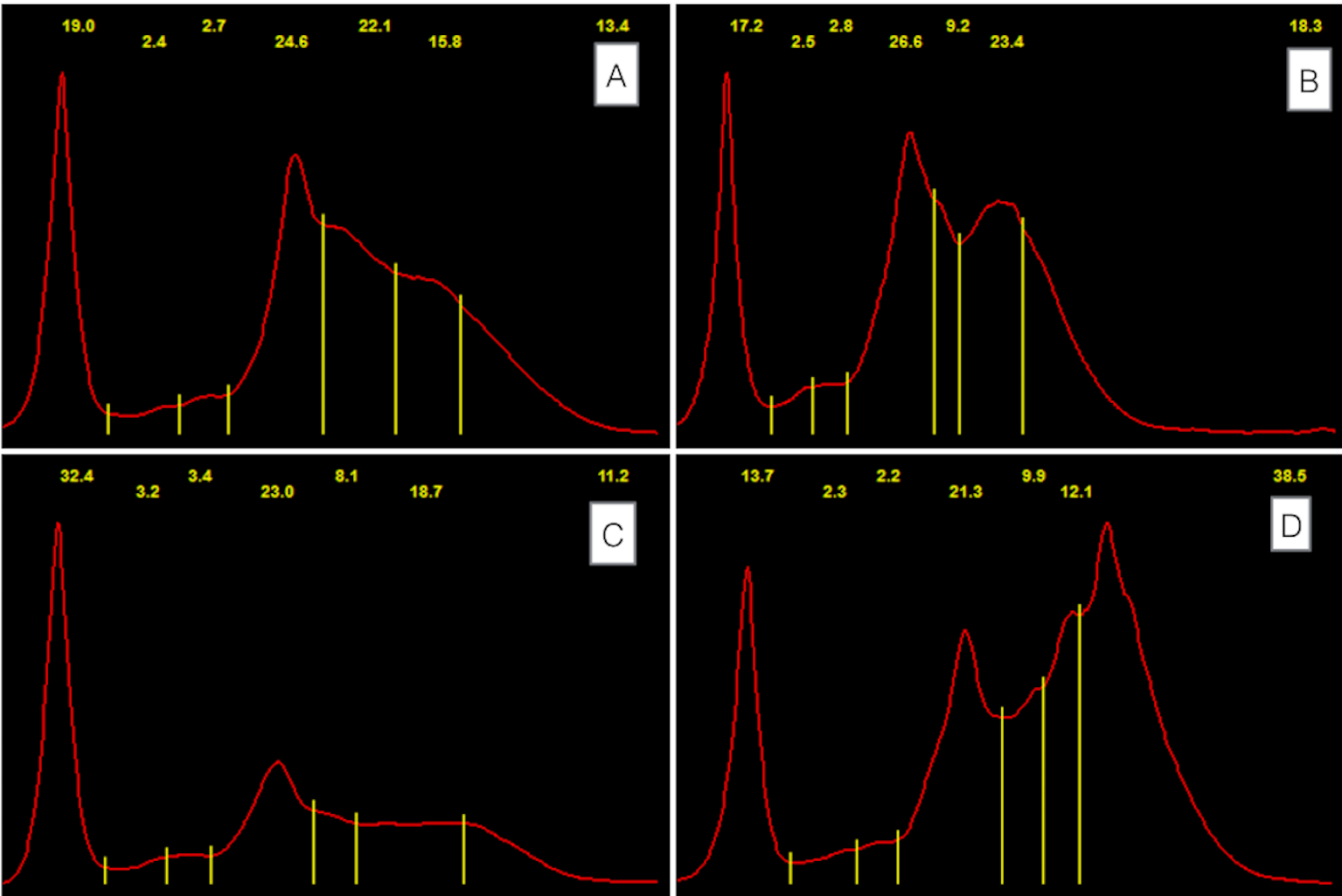
Serum agarose gel tracings from white rhinoceros. (A) Typical serum agarose gel electrophoresis pattern from a healthy adult white rhinoceros (sample 15). TSP 85 g/L, A/G 0.22. (B) Adult male (sample number 54) with extensive fight wounds (acute) has hypoproteinemia with hypoalbuminemia and decreases in all globulin fractions apart from γ -globulins. TSP 53 g/L, A/G 0.22. (C) Female calf (sample 80) post-colic surgery (acute) with aspiration pneumonia has a severe hypoproteinemia with marked decreases in α2-, β- and γ-globulins. TSP 48 g/L, A/G 0.46. (D) Adult male (sample 64) with multiple bullet wounds (chronic) has protein concentrations for all fractions within reference intervals, but the electrophoretogram tracing shows a relative polyclonal gammopathy of the β-2 and γ -globulins. TSP 78 g/L, A/G 0.21. Fractions from left to right are: albumin, α1a, α1b, α2, β1, β2 and γ -globulins. Numbers in yellow script indicate percentage of each fraction. TSP: Total serum protein, A/G: Albumin: globulin ratio, SPE: Serum protein electrophoresis.

Seven protein fractions were identified: albumin, α1a, α1b, and α2 globulins, β1 and β2 globulins and γ-globulins. A typical electrophoretogram from a healthy white rhinoceros is shown in Fig 1A. Intra- and inter-gel imprecision are shown in Table 2. Values for Rf for each fraction, as an indication of the precision and location of fraction gating, are also presented in Table 2.

**Table 2:** Estimates of imprecision and Rf values for different protein fractions in white rhinoceros serum on the Interlab Pretty electrophoresis platform.

Protein fraction	Intra-gel CV (13 runs)	Inter-gel CV (8 runs)	Rf
Albumin	2.8%	6.5%	1.0 (1.0–1.0)
α1a	9.8%	11.0%	0.86 (0.82–0.88)
α1b	9.6%	11.3%	0.77 (0.71–0.81)
α2	2.7%	5.9%	0.64 (0.61–0.68)
β1	11%	20.0%	0.51 (0.46–0.58)
β2	8.9%	19.8%	0.40 (0.35–0.48)
γ	10.7%	12.3%	0.17 (0.14–0.20)

Rf: mean (and range)

#### Mass spectrometry

In total, 78 proteins were identified with a protein identification probability of 100% (S2 Table). Electrophoretic migration patterns have been reported for 22 of these for several species (including the horse, another Perissodactyl) which are shown in Table 3. Regarding the potential α1b band, seven of the identified proteins have been described to migrate to the α1 fraction, 11 to the α2 fraction and four to the β fraction; and in the potential α2 band, five proteins have been reported to migrate to α1, 13 to α2 and nine to β [12–15, 28–30]. The majority of proteins identified in both bands were therefore α-globulins, and this was taken as confirmation that these bands represented α rather than β fractions.

**Table 3:** Proteins identified in the candidate α1b and α2 bands, with their reported electrophoretic migration in other species, as previously reported [12–15, 28–30].

Protein name, species of origin	Uniprot accession number	In candidate α1b band	In candidate α2 band	Reported migration
Serum albumin, *Homo sapiens*	ALBU_HUMAN	Yes	Yes	Albumin (cat, [28–30], dog [15], human [13], horse [14]), α1 (cat [28, 29], dog [15], human [13], horse [14])
Serum albumin, *Equus caballus*	ALBU_HORSE	No	Yes	Albumin (cat [28–30], dog [15], human [13], horse [14]), α1 (cat,[28, 29], dog [15],human [13], horse [14])
Alpha-1-antitrypsin, *Equus caballus*	B5BV07_HORSE	Yes	No	α1 (cat [29], human [13])
Alpha-1B-glycoprotein, *Equus caballus*	F6VJR6_HORSE	Yes	No	α1 (cat [28, 29])
Inter-alpha-trypsin inhibitor heavy chain 2, *Equus caballus*	F7CTJ3_HORSE	Yes	Yes	α1 (cat [28])
Kininogen 1, *Equus caballus*	F7C0D9_HORSE	Yes	Yes	α1 (dog [15]), α2 (cat [28])
Vitamin D binding protein, *Equus caballus*	F6T0P6_HORSE	Yes	No	α1 (cat [28], dog [15]), α2 (human [13])
Serpin family C member 1/ antithrombin III, *Equus caballus*	F7CYR1_HORSE	Yes	Yes	α1 (dog [15]), α2 (cat [28]), β (cat [28])
Alpha-2 macroglobulin, *Equus caballus*	F6R942_HORSE	Yes	Yes	α2 (cat [28–30], human [13], horse [14])
Ceruloplasmin, *Equus caballus*	F6PQ46_HORSE	Yes	Yes	α2 (cat [28, 29])
Haptoglobin, *Equus caballus*	F6XWM5_HORSE	Yes	Yes	α2 (cat [28–30], human [13], horse [14])
Angiotensinogen, *Equus caballus*	F6W4R2_HORSE	Yes	No	α2 (cat [29])
Clusterin, *Equus caballus*	CLUS_HORSE	Yes	Yes	α2 (cat [28])
Alpha-2 glycoprotein, *Equus caballus*	F7CAB8_HORSE	Yes	Yes	α2 (cat [28])
Serotransferrin, *Equus caballus*	F6ZEH8_HORSE	Yes	Yes	α2 (horse [14]), β (cat [28], horse [14])
Hemoglobin subunit beta, *Ceratotherium simum*	HBB_CERSI	Yes	Yes	α2 (cat [28]), β (cat [28])
Fibronectin, *Equus caballus*	F7CN05_HORSE	No	Yes	α2 (human [13]), β (cat [28], human [13])
Serotransferrin, *Homo sapiens*	TRFE_HUMAN	No	Yes	α2 (cat [28]), β (cat [28, 29], dog [15], human [13])
Apolipoprotein B, *Ceratotherium simum*	G5CW76_CERSI	No	Yes	α2 (cat [29]), β (cat [29])
Apolipoprotein B, *Equus caballus*	F6YCW8_HORSE	No	Yes	α2 (cat [29]), β (cat [29])
Hemoglobin subunit alpha, *Ceratotherium simum*	HBA_CERSI	Yes	Yes	β (cat [28])
Hemopexin, *Equus caballus*	F6X1I8_HORSE	No	Yes	β (cat [28, 29], dog [15], human [13])

### Reference intervals

One individual was excluded due to suspected dehydration, as described above. Reference intervals were calculated from 49 healthy white rhinoceros. Descriptive statistics, statistical methods and the RIs derived for this population are presented in Table 4. No difference was found between sexes for any of the fractions.

**Table 4:** Serum protein reference intervals for the white rhinoceros.

Analytes (Units)	Number of reference individuals	Mean	SD	Median	Min	Max	RI	LRL 90% CI	URL 90% CI	Distribution	Method
TSP (g/L)	49	89	7	91	75	111	76–111	75–78	99–111^1^	NG	NP
Albumin (g/L)	49	18	4	19	9	27	10–27	8–12^1^	25–28	G	R
α1a (g/L)	49	2.4	0.4	2.5	1.6	3.2	1.6–3.2	1.6–1.8	3.0–3.2	NG	NP
α1b (g/L)	49	2.6	0.5	2.6	1.7	3.6	1.7–3.6	1.5–1.9	3.4–3.7	G	R
α2 (g/L)	49	21.4	2.6	21.7	16.4	27.9	16.1–26.6	15.2–17.2	25.4–27.71^1^	G	R
β1 (g/L)	49	12.2	2.8	12.7	5.2	18.0	6.6–18.2	5.4–8.0^1^	17.0–19.4^1^	G	R
β2 (g/L)	49	17.2	4.1	16.6	11.8	31.4	11.8–30.4	11.8–12.2	23.9–31.4^1^	NG	NP
γ (g/L)	49	15.5	3.1	15.3	10.5	23.9	10.4–23.1	9.9–11.1	21.3–25.1^1^	NG	T, R
Total globulins (g/L)	49	71	7	71	59	89	60–87	58–62	83–91^1^	NG	T, R
A/G	49	0.26	0.07	0.26	0.11	0.41	0.12–0.39	0.10–0.15	0.36–0.42	G	R
Albumin (%)	49	20.2	4.2	20.4	9.7	29.2	11.9–29.0	10.3–13.8^1^	27.3–30.8^1^	G	R
α1a (%)	49	2.7	0.4	2.8	1.8	3.6	1.8–3.6	1.8–1.9	3.3–3.6	NG	NP
α1b (%)	49	2.9	0.5	2.9	2.0	3.8	1.9–3.9	1.8–2.1	3.7–4.1	G	R
α2 (%)	49	24.0	2.5	23.9	19.3	29.5	19.3–29.4	18.5–20.1^1^	28.3–30.6^1^	G	T, R
β1 (%)	49	13.7	3.0	14.3	6.7	20.3	7.8–20.3	6.5–9.2^1^	18.8–21.4^1^	G	R
β2 (%)	49	19.3	4.2	18.6	12.8	33.8	12.7–29.5	11.9–13.7	27.0–32.4^1^	NG	T, R
γ (%)	49	17.1	3.1	16.8	13	26.9	13.0–26.2	13.0–13.3	21.9–26.9^1^	NG	NP
Total globulins (%)	49	79.7	4.3	79.6	70.8	90.3	70.8–88.3	69.2–72.4	86.4–90.4^1^	G	R

NG indicates a non-Gaussian distribution; G, Gaussian; T, Box-Cox transformation of the data; R, robust method; NP, non-parametric method; RI, reference interval; LRL, lower reference limit; URL, upper reference limit; A/G, albumin: globulin ratio

^1^ CI to RI ratio exceeds 20%

### Comparison of healthy to injured animals

Results of 32 samples from 30 white rhinoceros with tissue trauma were compared to results from 49 animals in the healthy group. As shown in Table 5, TSP, albumin, all α-globulins, β1-globulin and total SPE globulin concentrations were lower in injured animals as a group. The proportions of α2- and β1-globulins were also lower, but proportions of γ-globulins were higher. The most common abnormalities in injured animals (all) were hypoproteinemia, hypoglobulinemia, and decreased α2- and β1-globulin concentrations. No injured animals showed an increase in α- or β1-globulin concentrations or α2-globulin proportions. Results were similar when comparing subgroups with acute and chronic injuries to the healthy group. Around half of animals with chronic wounds had decreased β2 and total globulin concentration; a third had low γ-globulin concentrations and high albumin: globulin ratios (A/G). SPE tracings from three of the injured animals are shown in Fig 1B–1D; with corresponding descriptions of the tracings in the legend for Fig 1.

**Table 5:** Comparison of results for total serum protein, albumin and globulin fractions for healthy and injured white rhinoceros.

Analyte (Units)	Mean ± SD or median (IQR) for healthy group	Mean ± SD or median (IQR) for injured group (all)	Median (IQR) for acute group	Median (IQR) for chronic group	Injured (all) different from healthy group	Acute/ chronic different from healthy group	Frequency of injured animals (all) with results below *and above* the RI	Frequency of acute with results below *and above* the RI	Frequency of chronic with results below *and above* the RI
TSP (g/L)	91 (85–93)	79 (66–85)	75 (59–85)	80 (71–86)	Yes (*P*<0.0001)	Chronic (*P* = 0.0003); Acute (*P* = 0.0003)	41%; *0%*	35%; *0%*	54%; *0%*
Albumin (g/L)	18 ±4	14 ±4	15 (11–19)	12 (9–15)	Yes (*P*<0.0001)	Chronic (*P*<0.0001); Acute (*P* = 0.0141)	25%; *0%*	29%; *0%*	23%; *0%*
α1a- globulins (g/L)	2.4 ±0.4	1.9 ±0.5	2.0 (1.5–2.1)	2.1 (1.3–2.4)	Yes (*P*<0.0001)	Chronic (*P* = 0.0019); Acute (*P* = 0.0012)	31%; *0%*	35%; *0%*	31%; *0%*
α1b- globulins (g/L)	2.6 ±0.5	2.0 ±0.5	2.0 (1.8–2.3)	2.0 (1.6–2.5)	Yes (*P*<0.0001)	Chronic (*P* = 0.002); Acute (*P* = 0.002)	22%; *0%*	29%; *0%*	15%; *0%*
α2- globulins (g/L)	21.4 ±2.5	15.8 ±4.5	16.0 (10.7–19.3)	16.8 (13.3–18.9)	Yes (*P*<0.0001)	Chronic (*P*<0.0001); Acute (*P* = 0.001)	50%; *0%*	47%; *0%*	54%; *0%*
β1- globulins (g/L)	12.2±2.8	8.8 ±3.7	8.0 (4.7–9.2)	9.3 (6.7–12.7)	Yes (*P*<0.0001)	Chronic (*P* = 0.0078); Acute (*P* = 0.003)	34%; *0%*	24%; *0%*	38%; *0%*
β2- globulins (g/L)	16.6 (14.2–19.4)	17.3 (10.2–21.0)	12.4 (8.1–18.6)	18.3 (14.2–21.1)	No	No	28%; *9%*	18%; *12%*	46%; *8%*
γ-globulins (g/L)	15.3 (13.0–17.1)	15.3 (11.1–17.4)	14.0 (7.9–16.9)	16.8 (11.8–20.0)	No	No	16%; *9%*	6%; *6%*	31%; *8%*
Total globulins (g/L)	71 ±7	60 ±17	57.6 (40.8–68.1)	67.9 (57.8–72.1)	Yes (*P* = 0.007)	Acute (*P* = 0.0023)	38%; *3%*	29%; *0%*	54%; *7%*
A/G	0.26 (0.18–0.27)	0.22 (0.17–0.30)	0.30 (0.21–0.45)	0.19 (0.18–0.22)	No	Chronic (*P* = 0.0015)	6%; *13%*	6%; *0%*	8%; *31%*
Albumin (%)	20.2 ±4.2	19.4 ±6.8	22.1 (5.4–29.0)	16.1 (13.6–17.9)	No	Chronic (*P* = 0.0012)	9%; *13%*	12%; *0%*	38%; *31%*
α1a- globulins (%)	2.8 (2.4–3.0)	2.6 (2.1–3.1)	2.8 (2.2–3.1)	2.4 (2.0–3.0)	No	No	13%; *9%*	18%; *0%*	0%; *15%*
α1b- globulins (%)	2.9 ±0.5	2.8 ±0.7	2.7 (2.4–4.1)	2.7 (2.2–2.9)	No	Chronic (*P* = 0.0229)	9%; *13%*	18%; *0%*	0%; *31%*
α2- globulins (%)	23.9 (22.2–25.5)	21.7 (20.1–24.1)	23.3 (21.6–24.3)	21 (19.2–23.4)	Yes (*P* = 0.0028)	Chronic (*P* = 0.0047)	16%; *0%*	24%; *0%*	8%; *0%*
β1- globulins (%)	14.3 (12.2–15.5)	11.2 (9.8–13.5)	10.7 (9.4–13.4)	11.9 (10.1–14.8)	Yes (*P* = 0.0065)	Acute (*P* = 0.0093)	6%; *3%*	12%; *0%*	15%; *0%*
β2- globulins (%)	18.6 (16.8–21.7)	21.7 (16.1–25.5)	16.5 (14.6–24.6)	22.1 (19.0–25.3)	No	Chronic (*P* = 0.0235)	3%; *9%*	0%;,*18%*	8%; *8%*
γ-globulins (%)	16.8 (14.3–18.3)	19.5 (15.2–22.0)	16.6 (14.2–20.9)	20.7 (18.4–24.4)	Yes (*P* = 0.0294)	Chronic (*P* = 0.0016)	9%; *9%*	0%; *18%*	23%; *8%*
Total globulins (%)	79.7 ±4.3	80.6 ±6.8	76.8 (68.7–82.7)	83.9 (82.1–86.4)	No	Chronic (*P* = 0.0013)	13%; *9%*	0%; *12%*	31%; *8%*

Results are presented as mean ± SD for data with a Gaussian distribution, and as mean (interquartile range) for non-Gaussian data.

A/G, albumin: globulin ratio; IQR, interquartile range; RI, reference interval

Data are presented for all injured animals and for acute and chronically injured sub-groups.

## Discussion

### Electrophoresis

The estimates of imprecision for the Pretty Interlab are higher than those reported for other species on other platforms and also higher than those reported by this manufacturer for human serum [20, 31–33]. Both intra- and inter-gel imprecision was less than 8% for all fractions in these publications. In our study, the separation of fractions was highly standardized. Variation in fraction gating is therefore unlikely to have contributed significantly to the imprecision. The high inter-gel imprecision found in this study may be explained partly by changes in protein fractions during aliquot refrigeration for up to 48 hours as this has been reported to occur in equine, caprine and bovine serum [34–36]. This does not, however, explain the high imprecision found within one gel. This may be due to a matrix effect associated with white rhinoceros serum, agarose gel quality or factors inherent to the automated system itself. As this particular platform has not been used in publications concerning other animal species, the source of the error is difficult to determine. Due to this high imprecision, small changes in protein fractions that might be seen in serial measurements from one individual should not be over interpreted as being clinically relevant.

A major preanalytical limitation of this study is that serum samples were frozen at different temperatures and for varying periods of time before analysis. This may have resulted in differential changes in protein fractions and affected the accuracy and interpretation of the results reported here. However, due to the opportunistic sampling required for this study, it was not possible to standardize the duration and conditions for storage of all samples.

The results of the agarose gel electrophoresis differ from published cellulose acetate SPE data for white rhinoceros with heterogeneity in the concentrations for each fraction between this study and other studies as well as between cellulose acetate membrane studies [6–8]. Differences between agarose and cellulose acetate matrices probably only play a minor role [20]. Heterogeneity in agarose gel test systems, specifically the buffers and stains may affect results [13]. Variation in the method of gating of the fractions probably has a much greater effect. This is substantiated by the variation seen within the cellulose acetate studies [6–8]. Variations in naming and values of fractions due to the “human effect” has been reported in cats, horses and birds, for example [32, 37, 38]. Using a standardized method, as was done in our study, to separate fractions decreases imprecision and improves reliability and has been used successfully in other studies, three of which involved horses [22, 32, 37, 39, 40]. Naming of globulin fractions generally proceeds according to convention but can also vary for a single species between different studies and lacks standardisation [32, 41]. A recommendation for SPE studies would be for investigators to describe the method of gating, present a typical tracing as an example, and use a standardized method for gating all samples from a specific species.

Other methods can be used in order to more accurately identify the fractions. These are based on the identification of specific proteins within fractions and include exchange chromatography, special stains, and mass spectrometry [14, 15, 28, 42]. For our study, the designation of the third, large globulin fraction as α2- rather than β1-globulin was confirmed by the finding, through proteomic analysis, that the majority of proteins within this fraction have been described to have an α-globulin migration in other species, including the horse. However, as can be seen in Table 3, many proteins do not migrate solely to one fraction and have wider migration distributions than conventionally assumed. In addition, protein migration is not identical across species and a protein may be present in different fractions in different animals. This could be due to variability in glycosylation patterns, the presence of protein fragments, or different isoforms [13, 28].

The width of the CIs of the reference limits exceeded the width of the RI by more than 20% for several protein fractions particularly for the upper reference limits. This illustrates a high degree of uncertainty around these reference limits. This is a reflection of the small sample size, and care should be taken when interpreting results falling near these reference limits as either absolutely normal or abnormal [25].

The means of determining the health status of the reference sample population in this study were limited, and “healthy” rhinoceros may have been suffering from conditions not detected during a clinical examination. However, white rhinoceros suffer from few clinical diseases, especially in free-range conditions. It is recognized that these animals were not parasite-free, as normal tick loads were observed, but were not considered a health issue. Additionally, the effect of immobilization (both drugs and stress of the procedure), on changes in serum proteins in the white rhinoceros is unknown. However, both the healthy and injured white rhinoceros were immobilized using the same drug combinations, other than the injured calves. Although a limitation, immobilization would likely have had a similar effect on both the healthy and injured rhinoceros. Immobilized white-tailed deer were shown to have lower albumin and total protein concentrations compared to physically restrained deer, although the differences were not clinically significant. These changes were speculated to be due to a drug related increase in capillary permeability resulting in haemodilution and a relative decrease in serum proteins [43].

White rhinoceros have high concentrations of globulins and low concentrations of albumin, with a resulting lower A/G compared to other species, including the horse. This appears to be the case for both free-ranging and captive populations. Reference intervals for captive white rhinoceros provided by the Species360 database for captive wildlife are: total protein 55–99 g/L (166 individuals), albumin 11–36 g/L (147 individuals) and globulin 30–77 g/L (153 individuals) (analytical methods not described) [44]. Globulin, and thus total protein, concentrations in free-ranging white rhinoceros appear to be approximately 10 g/L higher than in captive animals.

The bulk of the globulins consist of α2-, β2- and γ-globulins. Since these latter two fractions contain the various classes of antibodies, it appears that free-ranging white rhinoceros may naturally have high concentrations of immunoglobulins. This would need to be confirmed by immunoelectrophoresis if rhinoceros-specific antibodies become available in the future. The physiology behind this is not clear. Immunoglobulins are produced by B-lymphocytes. White rhinoceros peripheral leukocyte counts are neutrophil, rather than lymphocyte-dominated, and absolute lymphocyte counts are similar to those reported for domestic horses, donkeys and black rhinoceros [9, 44–48]. White rhinoceros lymphocytes did not exhibit increased proliferative responses to mitogen or antigen stimulation compared to Indian and Sumatran rhinoceros although lymphocyte responsiveness was superior to those of black rhinoceros [49]. Increased lymphocyte numbers or reactivity therefore do not appear to be a major reason for the high immunoglobulin concentrations in the white rhinoceros. The higher globulin concentrations seen in free-ranging versus captive animals may be related in part to the presence of ecto- and endoparasites. Various species of ticks, as well as gastrointestinal bot fly larvae, strongylids and pinworms have been identified in free-ranging white rhinoceros [50]. In addition, a high prevalence (36–49%) of non-pathogenic *Theileria bicornis* infections have been reported in this species [51, 52]. Neutralizing IgG2 antibodies have been shown to play a role in immunity to *Theileria* in cattle [53]. Horses with equine piroplasmosis were found to have an increase in γ-globulins compared to healthy controls due to the humoral immune response [54].

The two major proteins migrating to the α2-globulin fraction are the positive acute phase proteins haptoglobin and α2-macroglobulin. Haptoglobin is a hemoglobin binding protein that also has anti-oxidant and immunomodulatory roles, while α2-macroglobulin is a protease inhibitor [55, 56]. Direct measurement of haptoglobin concentrations in the white rhinoceros would shed light on whether this protein contributes significantly to the large α2-globulin present in this species. Further investigation of the acute phase response is also warranted.

Serum proteins play a major role in blood colloid oncotic pressure (COP) with albumin exerting a major effect. Even though albumin has been reported to exert twice the amount of oncotic pressure as globulins in humans and 4.4 times as much as globulins in cattle, γ-globulins themselves contribute significantly to COP [57, 58]. Increased concentrations of the latter cause a compensatory decrease in production of albumin in order to maintain oncotic pressure [59]. This may be the reason for the low albumin concentrations in the white rhinoceros, in compensation for the high globulin concentrations. A study investigating COP in healthy cattle, dogs, horses and rats found that samples with a low A/G had a lower COP than samples with a higher A/G, when TSP was the same [58]. Furthermore, in a study examining COP in wildlife species, the white rhinoceros was found to have a COP of 21.4 ± 3.9 mmHg, towards the lower end of the range (from 15 mmHg in steenbok to 64 mmHg in blue wildebeest; other hindgut fermenters: African elephant 47.0 ± 12.9 mmHg, Burchell’s zebra 40.0 ± 16.1 mmHg, Mountain zebra 25.8 ± 13.1 mmHg) [57]. These findings are interesting in that they suggest an overcompensation of the decrease in albumin, since COP is low normal. Albumin is also a negative acute phase protein, and the acute phase response may be another mechanism contributing to low concentrations of this protein in this species [56].

#### Comparison of healthy to injured animals

Most white rhinoceros with tissue trauma did not exhibit a typical acute phase reaction. Hypoproteinemia with hypoalbuminemia and decreases in α2- and β1 globulins were the most common changes. These changes are associated with the presence of wounds and tissue trauma in these animals. Similarities with human wound patients can be drawn (the phrase “human wound patients” hereafter describes patients with combat injuries, burn wounds, diabetic ulcers and pressure sores, among other causes of wounds). Human patients with wounds can lose up to 100 g of protein through wound exudation per day [60]. Patients with second-degree burn wounds covering 20% of body surface area were found to lose the equivalent of their entire serum protein mass over 24 hours, leading to the development of hypoproteinemia and hypoalbuminemia over the first 48 hours post injury [61]. Haptoglobin and various classes of immunoglobulins have been identified in wound exudate [61, 62]. Due to the catabolic state associated with trauma, the loss of protein through wound exudation, and the increased protein demand by healing wounds, dietary protein supplementation is considered vital for human wound patients [60]. Protein requirements are higher for patients with larger, deeper wounds, and those with wound infections, deep tracts, and high amounts of non-viable tissue. It can take up to four weeks to normalize serum protein concentrations in these patients, even when fed a high protein diet [60]. As the injured animals did not receive additional protein in their diet, it is unknown whether the hypoproteinemia could have been corrected nutritionally. Acute or chronic inflammatory responses based on measurement of TSP and globulin concentrations can be masked by the increased protein demand. This can be seen in the tracings in Fig 1B and 1D –these individuals have relatively high β2 and γ-globulin fractions in comparison to other proteins, indicative of chronic inflammation. The calf shown in Fig 1C displays a severe hypoproteinemia and hypoglobulinemia, possibly due to lack of adequate nutrition and immune suppression.

A limitation of this comparison is that white rhinoceros of different life stages, under varying husbandry and possibly immobilized using different drug dosages and combinations were included in the group with tissue trauma while the healthy group consisted only of adult animals. However TSP, albumin and globulin concentrations were not found to differ between white rhinoceros adults, sub-adults, or calves in another study [3].

## Conclusion

The high serum globulin concentration of the white rhinoceros is due to high α2-, β2- and γ-globulins. This suggests a very active humoral immune system: possibly an adaptation to the high prevalence of blood-borne and other parasites. The contribution of haptoglobin to the high concentrations of α2-globulins deserves further study. A full serum proteome analysis from a variety of healthy and sick animals will provide more comprehensive data and should be further researched. White rhinoceros with tissue trauma display protein changes typical for wound patients and an acute phase response is not evident. Additional studies evaluating the effect of dietary protein supplementation should be considered in these patients.

## Supporting information

S1 Text Methods. Detailed liquid chromatography–mass spectrometry method description.
(DOCX)

S1 Table Rhinoceros data. Raw SPE data and clinical information for healthy and injured rhinoceros.
(XLSX)

S2 Table Proteomic results. Detailed information for the proteins identified in the alpha1b and alpha2 bands.
(XLSX)
