# Supplementary material for: Serum protein electrophoresis in healthy and injured southern white rhinoceros (Ceratotherium simum simum)
Source: PLoS One. 2018 Jul 25;13(7):e0200347. doi: 10.1371/journal.pone.0200347 (PMC6059428; doi:10.1371/journal.pone.0200347)
Supplement: S1 Text — Detailed liquid chromatography–mass spectrometry method description. (DOCX) [file pone.0200347.s001.docx]

**Supplemental methods section (Methods S1)**

Peptide extraction

The excised agarose gel bands were destained with 10% acetonitrile (Fluka, Honeywell, Bucharest, Romania) in 100 mM Tris pH 8 before reduction with 2 mM triscarboxyethyl phosphine (TCEP; Fluka) in 100 mM NH_4_HCO_3_ for 15 minutes at room temperature with agitation. Excess TCEP was removed and the gel pieces washed twice with 100 mM NH_4_HCO_3._ Proteins were digested by rehydrating the gel pieces in trypsin (Pierce, Thermo Fisher Scientific, Waltham, MA, USA) solution (20 ng/μL) and incubating at 37 °C overnight. Peptides were extracted from the gel pieces once with 50 μL water and once with 50% acetonitrile. The samples were dried down and resuspended in 30 μL of an aqueous solution containing 2% acetonitrile and 0.1% formic acid (FA). Residual digest reagents were removed using an in-house manufactured C18 stage tip (Empore Octadecyl C18 extraction discs; Supelco, Sigma-Aldrich, Johannesburg, South Africa). The samples were loaded onto the stage tip after activating the C18 membrane with 30 μL methanol (Sigma-Aldrich) and equilibration with 30 μL of an aqueous solution containing 2% acetonitrile and 0.05% TFA. The bound sample was washed with 30 μL of an aqueous solution containing 2% acetonitrile and 0.1% TFA before elution with 30 μL of an aqueous solution containing 50% acetonitrile and 0.05% TFA. The eluate was evaporated to dryness. The dried peptides were dissolved in an aqueous solution containing 2% acetonitrile and 0.1% FA for liquid chromatography-mass spectrometry analysis.

Liquid chromatography

Liquid chromatography (LC) was performed on a Thermo Scientific Ultimate 3000 RSLC (Thermo Fisher Scientific) equipped with a 2 cm x 100 μm C18 trap column and a 35 cm x 75 μm in-house manufactured C18 analytical column (Aeris C18, 3.6 μm; Phenomenex, Torrance, CA, USA). The solvent system was loading Solvent A consisting of 2% aqueous acetonitrile with 0.1% FA and Solvent B consisting of 100% aqueous acetonitrile. The samples were loaded onto the trap column using loading solvent at a flow rate of 15 μL/min from a temperature controlled autosampler set at 7°C. Loading was performed for 5 min before the sample was eluted onto the analytical column. Flow rate was set to 500 nL/minute and the gradient generated as follows: 2.0% -10% B over 5 min; 5% -25% B from 5-50 minutes using Chromeleon (Thermo Fischer Scientific) non-linear gradient 6, 25%-45% from 50-65 minutes, using Chromeleon non-linear gradient 6. Chromatography was performed at 50°C and the outflow delivered to the mass spectrometer through a stainless steel nano-bore emitter.

Mass spectrometry

Mass spectrometry (MS) was performed using a Thermo Scientific Fusion mass spectrometer (Thermo Fischer Scientific) equipped with a Nanospray Flex ionization source. Data was collected in positive mode with spray voltage set to 2 kV and ion transfer capillary set to 275°C. Spectra were internally calibrated using polysiloxane ions at m/z = 445.12003 and 371.10024. MS1 scans were performed using the orbitrap detector set at 120 000 resolution over the scan range 350-1650 with automatic gain control target at 3 E5 and maximum injection time of 45 ms. Data were acquired in profile mode. MS2 acquisitions were performed using monoisotopic precursor selection for ion with charges +2-+6 with error tolerance set to +/- 10ppm. Precursor ions were excluded from fragmentation once for a period of 30 s. Precursor ions were selected for fragmentation in HCD mode using the quadrupole mass analyzer with higher energy collisional dissociation set to 32.5%. Fragment ions were detected in the orbitrap mass analyzer set to 15 000 resolution. The automatic gain control target was set to 1E4 and the maximum injection time to 45ms. The data were acquired in centroid mode.

Protein identification

The raw files generated by the mass spectrometer were imported into SearchGUI (Compomics, Ghent, Belgium) and the X!Tandem algorithm was selected. Database interrogation was performed against a concatenated database created using the Uniprot (22) order Perissodactyla database and a contaminant database with semi-tryptic cleavage allowing for 2 missed cleavages. Precursor mass tolerance was set to 10 ppm and fragment mass tolerance set to 0.02 Da. Protein deamidation (NQ) and oxidation (M) was allowed as dynamic modifications. The msf file output from Proteome Discoverer was imported into Scaffold Q+ (version 4.4.6, Proteome Software Inc., Portland, OR) and additional validation performed. Proteins were considered to be positively identified if the protein identification probability was 100%.
